# Impact of breast cancer on prospective memory functioning assessed by virtual reality and influence of sleep quality and hormonal therapy: PROSOM-K study

**DOI:** 10.1186/s12885-018-4762-2

**Published:** 2018-09-03

**Authors:** Mylène Duivon, Joy Perrier, Florence Joly, Idlir Licaj, Jean-Michel Grellard, Bénédicte Clarisse, Christelle Lévy, Philippe Fleury, Sophie Madeleine, Nicolas Lefèvre, Géraldine Rauchs, Grégory Lecouvey, Florence Fraisse, Fausto Viader, Francis Eustache, Béatrice Desgranges, Bénédicte Giffard

**Affiliations:** 1 Normandie Université, UNICAEN, PSL Université, EPHE, INSERM, U1077, CHU de Caen, Neuropsychologie et Imagerie de la Mémoire Humaine, 14000 Caen, France; 2 Clinical Research Department, Centre François Baclesse, 14076 Caen, France; 3 Normandie Université, UNICAEN, INSERM, U1086 ANTICIPE, 14076 Caen, France; 4 Medical Oncology Department, CHU de Caen, 14000 Caen, France; 5 Cancer and Cognition Platform, Ligue Nationale Contre le Cancer, 14076 Caen, France; 6 Medical Oncology Department, Centre François Baclesse, 14076 Caen, France; 7 Normandie Université, UNICAEN, CIREVE, 14000 Caen, France; 8 Neurology Department, CHU de Caen, 14000 Caen, France; 9 Pôle des Formations et de Recherche en Santé, 2 rue des Rochambelles, CS-14032 Caen Cedex, France

**Keywords:** Prospective memory, Virtual reality, Sleep quality, Breast cancer, Hormonal therapy

## Abstract

**Background:**

Breast cancer (BC) is the most frequent cancer in women with more than 70% of BC patients being treated with hormonal therapy (HT). Among these patients, some report difficulties in remembering what they are supposed to do at the right moment, referring to prospective memory (PM). PM is essential for autonomy and medical adherence of patients, and requires an ecological assessment. Virtual reality, that recreates naturalistic environment, seems to be a promising method to evaluate PM. Several BC patients also report sleep disturbances. Given the role of sleep on memory consolidation, it is imperative to explore the influence of sleep quality on PM in BC patients treated with HT.

The purpose of PROSOM-K study is to assess PM functioning using virtual reality and sleep quality in BC treated or not with HT.

**Methods:**

PROSOM-K is a prospective study including post-menopausal BC patients ≤70 years old treated with radiotherapy (*n* = 25) or with radiotherapy and HT (*n* = 25), and healthy post-menopausal women (*n* = 25) matched for age and education. PM will be assessed using a virtual reality based task. Other cognitive functions and psychosocial factors will be assessed with validated questionnaires and neuropsychological tests. The study is divided in 3 sessions: a session of familiarisation with the virtual environment and the PM task: a day-time session during which participants learn intentions during the morning and recall them in the evening; and a night-time session during which participants learn intentions in the evening and recall them the following morning. Women will be monitored by wrist actigraphy; during the night-time session, objective sleep quality and quantity will be measured by polysomnography.

**Discussion:**

This is a novel study aiming to assess PM using virtual reality, coupled with the evaluation of other cognitive functions. Polysomnographic study of sleep will provide further information about architectural sleep disturbances in BC. Association between sleep architecture parameters and PM mechanism in BC women treated with HT will be described in detail. We expect our results will provide knowledge for patients and clinicians and further help to improve patient care and cognitive therapy.

**Trial registration:**

NCT03420105, registered: January 10, 2018.

**Electronic supplementary material:**

The online version of this article (10.1186/s12885-018-4762-2) contains supplementary material, which is available to authorized users.

## Background

Breast cancer (BC) is the most frequent cancer in women and its incidence is increasing [1]. Among these patients, some of them experience cognitive troubles and especially difficulties remembering what there are supposed to do at the right time or in the right place [2, 3]. These memory lapses concern prospective memory (PM) which refers to our ability to remember to accomplish intended tasks or actions at some predefined point in the future.

PM is divided into two components: prospective and retrospective. The first of these refers to the process of remembering that something has to be done at the appropriate time or event, while the second refers to the memory of what has to be done (action). Prospective component gathers two types of intentions: Time-Based (TB) intention referring to self-initiated retrieval of the action after a period of time has elapsed or at a certain time; and Event-Based (EB) intention when an event occurs and triggers the remembering of the action. Intention retrieval relies on two types of processes used in a Dynamic Multiprocess Framework: monitoring and spontaneous retrieval. Their use depends on various factors, for example, characteristics of intentions and ongoing tasks, and importance of realising the action. Monitoring refers to keeping an intention in mind while searching for the prospective component. Spontaneous associative retrieval process relies on automatic processes that bring a retrospective component into mind when a prospective component appears [4]. PM functioning involves various cognitive functions, such as episodic memory, executive functions, and working memory. Retrospective episodic memory is required for the encoding, retention and recall of the intention [5]. Executive functions are involved in planning the intention, inhibiting the ongoing task, and switching to realise the intended action. Working memory is required to keep the intention in mind between the recall and the realisation of the action, and the binding process induces a link between prospective and retrospective components creating a unitary representation of the intention in working memory [6]. PM is essential for daily living tasks and, in BC patients, PM is even more essential for medical adherence, autonomy, and return to social and professional life.

Despite recurrent complaints of patients about their PM, only a few studies have focused on PM deficits in BC [2, 3, 7–10]. Paquet and colleagues used the Memory for Intention Screening Test (MIST) [11], a common PM task, composed of four EB and four TB intentions to retrieve while completing a word puzzle in the laboratory. A naturalistic task had to be carried out at home 24 h later. The authorsdid not differentiate between EB and TB intentions, but their results revealed decreased performances of patients compared to healthy women [2]. Cheng and colleagues used two laboratory tasks, assessing TB and EB intentions separately. During these tasks, participants had to tap the desk at the target time or event. Results showed a deficit in EB intention retrieval in BC patients [3, 9, 10]. Altogether, results from these studies revealed an impairment of PM in BC patients. However, none distinguished between prospective and retrospective components, which limits the understanding of the impaired processes. In addition to PM, Cheng and colleagues [4–6] assessed general cognitive functioning with Mini Mental State Examination, Verbal Fluency Test, and Digit Span test. Results revealed a significant decrease of cognitive functioning in BC patients compared with healthy women, but the authors did not correlate neuropsychological scores with PM scores. Cognitive functions known to be involved in PM functioning must be evaluated and correlated with PM scores in order to better understand the PM deficits in BC patients.

Furthermore, laboratory PM tasks lack ecological validity. Virtual reality may be suitable to assess PM because it recreates naturalistic situations of daily life while maintaining experimental rigor [6] that is difficult to uphold in naturalistic PM tasks. Other factors associated with BC (depression, stress, anxiety, and fatigue) may affect both cognition and PM [2, 12], and should therefore be taken into account to deepen knowledge about factors implicated in PM deficits in BC patients.

Studies in BC revealed that 39.5% to 69% of patients encounter sleep disturbances, provoked or worsened by cancer and its treatments [13]. The majority of studies used self-report questionnaires and reported sleep disturbances in BC patients. Studies on patients treated for BC using wrist actigraphy to assess motor activity and sleep quality reported a decreased sleep quality. In cancer patients, very few studies have used polysomnography (PSG), the gold standard of objective assessment of sleep. Although results of sleep architecture (particularly percentages of sleep stages) differ between studies using PSG, they showed a deterioration of sleep quality in BC patients. Furthermore, one of the parameters of interest, the Slow Waves Sleep (SWS), involved in memory consolidation, seems to be decreased in cancer patients [14–16].

Sleep is well known to play a part on memory consolidation [17–19]. While consolidation of retrospective memories is well studied, few studies have been published on benefits of sleep in PM although mechanisms might be different. Scullin and McDaniel showed that remembering to execute an intention was improved after a 12 h sleep delay compared to a 12 h wake delay [19]. Diekelmann and colleagues demonstrated that subjects who slept during the early night (mostly composed of SWS), had better scores at intention execution two days later than subjects who had slept during the late night (mostly composed of Rapid Eye Movement sleep) [20]. Sleep, and especially SWS, seems to promote PM consolidation for simple tasks. Results on sleep benefits in more complex and ecological tasks need to be thorough, as well as sleep variables implicated in PM consolidation. In light of SWS involvement in memory consolidation, the link between sleep disturbances and PM deficits in BC patients need to be explored.

Among factors that may have an impact both on memory and sleep disturbances in BC patients, hormonotherapy seems particularly relevant. The majority of studies assessing impact of treatment on BC patients have focused on chemotherapy, while up to 70% of BC patients are treated with hormonal therapy (HT) [21]. Some studies have shown a deleterious impact of HT on patients' cognitive functions, especially memory [22]. Only one study has made an a posteriori analysis about the PM performances at MIST in BC patients treated or not with HT [7]. There were no significant differences between scores of these two groups, probably because components and EB/TB intentions were not differentiated. Furthermore, all patients were also treated with chemotherapy, a treatment known to have an impact on cognitive functioning. Thus, this study does not allow for a conclusive understanding of the impact of HT on PM. Few studies have been published about sleep disturbances in BC treated with HT. Additionally, these studies have mostly used self-report questionnaires about sleep quality and results are inconclusive [23]. Some studies reported insomnia complaints from patients treated with HT [24], while others did not [25].

Further studies are, therefore, needed to confirm the impact of HT on PM and sleep in BC patients. This also warrants to implement studies with ecological tasks taking into account the different components of PM coupled with complementary neuropsychological tests to understand processes impaired. Furthermore, no study has assessed the influence of sleep disturbances on PM in BC patients.

The purpose of PROSOM-K is to evaluate cognitive functions underlying PM impairment in BC patients using an ecological task and virtual reality. Further, we aim to assess sleep disturbances encountered by BC women treated or not with HT and their impact on PM functioning.

## Methods

### Study objectives

The primary objective of the PROSOM-K study is to assess PM performances in BC patients, in order to determine which components (prospective or retrospective), and types of intentions (EB or TB) are the most impaired in BC patients, and specify which cognitive processes (episodic memory, working memory, or executive functions) are particularly involved in PM decline in BC patients.

The secondary objectives are to:

- clarify the influence of sleep disturbances in BC on PM deficits, using various sleep quality assessments (polysomnography, actigraphy and self-report questionnaires), and by comparing PM scores in two different conditions, depending on the type of delay between encoding and retrieval of intentions: day-time and night-time
- assess the influence of HT on PM functioning by comparing PM scores between BC patients treated with HT and BC patients not treated with HT.

### Participants

The PROSOM-K study will include 25 BC patients treated with radiotherapy alone, 25 BC patients treated with radiotherapy and HT, and 25 healthy women. Patients will be recruited in Centre François Baclesse, a regional cancer centre in Caen (Normandy, France). PROSOM-K protocol will be proposed to eligible patients during a follow-up medical care with their oncologist. Healthy volunteer women, matched for age and years of education with BC patients, will be recruited from our laboratory. Once verified the eligibility criteria (see Table 1) by a physician, each participant will provide her written informed consent to be enrolled in PROSOM-K protocol.

**Table 1:** PROSOM-K inclusion and non-inclusion criteria

	Breast cancer patients	Healthy women
Inclusion criteria	1 year post-menopausal	
	Under 70 years of age	
	At least on level 3 (end of primary schools) of the Barbizet scale	
	French native speakers	
	Treated with surgery or radiotherapy for a non-metastatic breast cancer	Normal cognitive function with the Montreal Cognitive Assessment (MOCA) score ≥ 26
	Radiotherapy completed about 6 months prior to the study	–
Non-inclusion criteria	Neurological sequelae	
	Personality disorders and progressive psychiatric disorder	
	Drug use and/or heavy drinking	
	Treated with chemotherapy	History of cancer, excepting basal-cell carcinoma and carcinoma in situ of the uterine cervix
	Patient with a paraneoplastic syndrome	–
	Patient unable to perform cognitive tests	–
	Central nervous system primitive tumour or cerebral metastases	–
	Primitive cancer different from Breast cancer	–
	Metastatic cancer	–
	Cognitive disorders pre-existing to cancer diagnosis	–

### Virtual environment

The three-dimensional immersive environment has been designed and developed by the Interdisciplinary Centre for Virtual Reality (CIREVE) in Caen (Normandy, France). The environment is a virtual reproduction of the Memorial Museum in Caen. The immersive room (CAVE, Cave Automatic Virtual Environment) is composed of four wide screens for 3D stereoscopic projection: two laterals (9 × 3 m), one facial (4.80 × 3 m), and one on the floor (9 × 4.80 m). Participants will wear stereoscopic glasses with position sensors able to compute perspective in real time. Participants will be placed at the centre of the floor screen [see Additional file 1] and will move using a joystick that also allow them to project the fictional time and a map of the Memorial onto the screen.

### PROSOM-K procedure

Following the phase of inclusion, the experiment is divided into three sequential sessions: session 1 (familiarisation), day-time session, and night-time session [see Additional file 2]. The interval between two consecutive sessions is about one week, and the order of day-time and night-time sessions is counterbalanced between participants. During each session, participants will have to learn nine intentions and retrieve them in the virtual environment after a delay of 10 min for session 1, and about 12 h for the two other sessions. The lists of 9 intentions are composed of 3 TB intentions (e.g. at 12:11 ➔ go to the restaurant for the lunch), 3 linked EB intentions, i.e. with a strong link between the prospective cue and retrospective component (e.g. at the cafeteria ➔ buy a black coffee), and 3 no-linked EB intentions (e.g. at the child-care centre ➔ ask for a map of the Memorial).

**Session 1** allows for the familiarisation of participants with virtual reality use and PM task. This session will be performed either at 8:30 or at 18:30 to assess a potential effect of time on learning and PM performances. A guided-visit of the virtual Memorial Museum will be realised to teach participants how to navigate in the virtual environment, display the time and the map with the joystick, and locate every place and component of the environment. The visit will be followed by a phase of learning the intentions, and after a delay of 10 min, participants will go back to the virtual Memorial to retrieve the intentions.

**Day-time session** is divided into two parts. The first part will take place at 8:30, subjects will learn new intentions. The second part will take place at 19:00, during which participants will go into the virtual environment and retrieve the intentions.

**Night-time session** is also divided into two parts. The first part will begin at 17:30, PSG will be placed, and after a meal, women will learn new intentions. Participants will come the next day at 8:30 to have PSG removed, and recall intentions in the virtual Memorial.

### PM task

**Learning phase**

The PM task will begin with a phase of intentions learning. Each intention will be displayed during 10 s on a laptop screen and participants will have to read each of them out loud. Following every three intentions, a cued recall test of these intentions will be realised (e.g. “What will you have to do at 12:11?”). Any unrecalled intentions will be repeated once. After the presentation of all intentions, a cued-recall test will be realised and unrecalled or incorrectly recalled intentions will be repeated. This operation will be done with all unrecalled intentions, until all of them are correctly encoded. Finally, to ensure that all intentions are correctly encoded, they will be retrieved in a last global cued recall test. At the end of this learning phase, participants will have to say how many intentions they think they will remember during the virtual reality task in order to assess their metamnesic awareness.

**Retrieval phase**

After an interval of 10 min or 12 h, participants will enter the virtual Memorial for the retrieval phase. Intentions will be retrieved during an ongoing task that requires participants to focus their attention on this task and not on the intentions. The ongoing task consists in visiting the Memorial and going to every location indicated by a yellow rectangle on the map, to observe and memorise photographs that they will have to be recognised after the PM task. Every time participants will be in front of a photograph, the yellow rectangle of the emplacement will disappear from the map. Thus, participants will be able to know what photographs remain to be seen.

For TB intentions, a button on the flystick will allow participants to display the hour on the screen. When at a certain time or place, subjects will retrieve an intention, they will have to say out loud what they are supposed to do. Even if the time is up, or if only prospective or retrospective components are retrieved, women will have to express it. Participants will be immersed for about 15 min in a visit of the Memorial, to view and memorise all photographs and retrieve the intentions.

At the end of the virtual reality task, participants will have to say how many intentions they think they have retrieved, which will allow us to assess their metamemory ability, and a free recall test of all intentions will be realised. For unrecalled intentions, a cued recall test will be done and participants will have to say for EB intentions if they have noticed the prospective component in the environment.

Then participants will realise a recognition task of the photographs seen during the ongoing task. Thirty photographs will be displayed on a laptop screen among which 15 are present in the virtual Memorial. Participants will have to respond “YES” or “NO”. The order of the screening of photograph sets is counterbalanced between day-time and night-time sessions, and the total score will be 30 points. Finally, they will complete a debriefing, noting from 0 to 10 the logic of the link between prospective and retrospective components and the importance for them to realise each intention. Indeed, the importance for the participant to realise an intention will influence her motivation and the processes used to retrieve the intention, and thus the probability of realising the intended action.

### Outcome measures

#### PM task

During the learning of each of the 9 intentions (3 per type of intention, i.e. 3 linked EB, 3 no-linked EB, and 3 TB), a score [0–1] will be attributed per intention for the first cued recall (see Table 2). We will also evaluate the number of repetitions needed to correctly recall all intentions. The maximum total PM encoding score of each session will be 9 points, corresponding to the sum of the points attributed for each intention (1point * 9 intentions).

During the retrieval of the 9 intentions, a score [0–6] will be attributed for each intention corresponding to the points allocated for the prospective component [0–2], the retrospective component [0–2], and the associative component (i.e., the simultaneous retrieval of prospective and retrospective components) [0–2] (see Table 2). The maximum total PM retrieval score of each session will be 54 points, corresponding to the sum of the maximal scores attributed for each intention (6 points * 9 intentions).

**Table 2:** Scoring of each intention of the PM task

LEARNING SCORE [range]			
1 point	Intention is correctly recalled during the immediate cued recall [0–1]		
RETRIEVAL SCORES [range]			
	Prospective [0–2]	Retrospective [0–2]	Associative [0–2]
2 points	Action realised at the first passage in front of the cue or at the right time	Retrospective component is complete	Prospective and retrospective components are recalled together
1 point	Action realised at the second passage or at +/− 1 min	Retrospective component is incomplete (one element is incorrect or forgotten)	–
0.5 point	Action is realised at a subsequent passage or at +/− 2 min	–	–
0 point	Action is not realised	Retrospective component is not retrieved	Prospective and retrospective components are not recalled together

#### Complementary cognitive assessment

During each session, cognition will be assessed using neuropsychological tests (see Table 3). Global cognitive functioning will be assessed with the Montreal Cognitive Assessment (MoCA) [26], and the crystallised intelligence with the Mill Hill part B [27]. Retrospective episodic memory will be assessed with the French adaptation of Grober and Buschke’s procedure [28]. Working memory will be assessed using the Digit Span forward and backward test (Wechsler Adult Intelligence Scale-III [29]), and the binding process will be assessed using a multimodal integration task [30]. During the multimodal integration task, participants have to mentally associate four coloured letters and the location of the cross of the same colour in a grid. Then a grid appears with a black letter, and participants have to indicate if the letter-location corresponds to the letter-location of the mental association. Executive functioning, especially planning, inhibition, shifting, and updating, which are known to be involved in PM functioning, will be assessed respectively with: the Zoo map test [31], the Stroop [32], the Trail Making Test [33], and the N-back task [34].

**Table 3:** Neuropsychological tests battery and questionnaires included in the PROSOM-K study, including outcomes measures

Domain assessed	Assessments	Outcome measures	Range
Neuropsychological tests			
Global functioning	MOCA [26]	Total number of correct responses	0–30
	Mill Hill [27]	Total number of correct responses	0–34
Episodic memory	RL/RI-16 [28]	Immediate free recall score	0–48
		Immediate total recall score	0–48
		Delayed free recall score	0–16
		Delayed total recall score	0–16
Working memory	Digit span forward and backward [29]	Total number of correct trials forward	0–16
		Total number of correct trials backward	0–16
	Multimodal integration task [30]	Total number of correct responses	0–20
Executive functions	Zoo map test [31]	Profile score	0–4
	Stroop [32]	interference–colour (time)	≥0 s
	TMT [33]	TMT B–TMT A (time)	≥0 s
		Perseverative errors TMT B (number)	≥0
	N-back [34]	Total number of correct responses	0–48
Questionnaires			
Cognitive self-assessment	Fact-Cog [35]	PCI/PCA/QOL/Oth	0–72/0–28/0–16/0–16
	PRMQ [36]	Prospective score	8–40
		Retrospective score	8–40
Anxiety	STAI [39]	State score	20–80
		Trait score	20–80
Mood	BfS/BfS’ [45]	Total score / Total score	0–56 / 0–56
Depression	BDI [40]	Total score	0–39
Self-esteem	QSR [38]	Valence score	0–100%
		Certainty score	0–100%
Quality of life	FACT-G (patients) [37]	Total score	0–108
Fatigue and somnolence	VAS-F [43]	Energy score / Fatigue score	0–50 / 0–130
	KSS [44]	Total score	1–9
	MFI [41]	General fatigue score	4–20
	FACIT-F (patients) [42]	Total score	0–52
Sleep	Questionnaire of the past 24 h	Sleep efficiency ([total sleep time – total time in bed] x100)	0–100%
	PSQI [47]	Total score	0–21
	ISI [48]	Total score	0–28
	Circadian typology (Horne and Ostberg)	Total score	16–86
Virtual reality discomfort	Simulator sickness [46]	Nausea score	0–27
		Oculomotor score	0–21

*MOCA* Montreal Cognitive Assessment, *RL/RI-16* free recall / cued recall 16 items (French version of the Grober & Buschke procedure), *TMT* Trail Making Test, *FACT-cog* Functional Assessment of Cancer Therapy-Cognitive Function, *PCI* Perceived Cognitive Impairments, *PCA* Perceived Cognitive Abilities, *QOL* impact of PCI on Quality Of Life, *Oth* Others, *PRMQ* Prospective Retrospective Memory Questionnaire, *STAI* State-Trait Anxiety Inventory, *BfS/BfS’* Befindlichkeits-Skala, *BDI* Beck Depression Inventory, *QSR* Questionnaire of Self-Representations, *FACT-G* Functional Assessment of Cancer Therapy-General, *VAS-F* Visual Analog Scale of Fatigue, *KSS* Karolinska Sleepiness Scale, *MFI* Multidimensional Fatigue Inventory, *FACIT-F* Functional Assessment of Chronic Illness Therapy – Fatigue scale, *PSQI* Pittsburgh Sleep Quality Index, *ISI* Insomnia Severity Index

#### Standardised validated self-report questionnaires

During each session, women will complete self-report questionnaires to assess cognitive functioning and psychosocial factors (see Table 3). Cognitive functioning will also be assessed by self-report questionnaires (Functional Assessment of Cancer Therapy Cognitive Scale: FACT-Cog [35] and Prospective Retrospective Memory Questionnaire: PRMQ [36]).

Self-report questionnaires will be used to assess quality of life (Functional Assessment of Cancer Therapy-General: FACT-G [37]), self-esteem (Questionnaire of Self-Representations [38]), anxiety (State-Trait Anxiety Inventory [39]), depression (Beck Depression Inventory [40]), and fatigue (Multidimensional Fatigue Inventory [41], Functional Assessment of Chronic Illness Therapy-Fatigue: FACIT-F [42]). Scale of fatigue (Visual Analogue Scale to Evaluate Fatigue Severity [43]) and somnolence (Karolinska Sleepiness Scale [44]) will be proposed before the learning phase, as well as before and after the phase of intentions retrieval. Mood will be assessed during each session before intentions retrieval by a self-rating mood scale (the Zerssen Befindlichkeits-Skala [45]). Virtual reality discomfort will be assessed by the simulator sickness questionnaire [46] during each session after the virtual reality based task.

#### Sleep assessment

Sleep quality and quantity, circadian typology and insomnia symptoms will be assessed using self-report questionnaires: questionnaire of the past 24 h (routinely used in our research unit, to assess sleep efficiency and sleep quality of the night before the session of familiarisation), Pittsburgh Sleep Quality Index [47], Insomnia Severity Index [48], and circadian typology (Horne and Ostberg) [49]. Every day between the first and the last sessions, participants will fill out a sleep diary to subjectively assess sleep quality and duration, including hours of bedtime, sleep quality, number and duration of nocturnal awakenings and naps.

During all the protocol, participants will wear an actigraph (MotionWatch 8, camNtech) on their non-dominant wrist. Actigraphy will give information related to sleep/activity rhythm, but also related to sleep quality on a longer duration than PSG (around two weeks). The following variables will be extracted: sleep onset latency, total sleep time, sleep efficiency ([actual sleep time – time in bed] x 100), number of nocturnal awakenings after sleep onset. Finally, it will allow to check whether participants took a nap or not during the day-time session or kept awake during the night-time one.

For the night-time session, sleep of participants will be recorded by ambulatory PSG (the gold standard evaluation of sleep quality) at home, in order to assess sleep onset latency, total sleep time, sleep efficiency, relative percentages of sleep stages, sleep efficiency, number of nocturnal awakenings after sleep onset. Twenty electroencephalography (EEG) electrodes will be placed over the scalp, over prefrontal (FP1/FP2), frontal (F3/F4/F7/F8/Fz), central (C3/C4/Cz), temporal (T3/T4), parietal (P3/P4/Pz), and occipital (O1/O2) sites, according to the international 10–20 system; using Ag/Au electrodes with a vertex ground and a bi-mastoids reference. The impedance for all electrodes will be kept below 5 kΩ. The hardware EEG filter band pass will be 0.15–121 Hz and the sample rate will be 256 Hz. Two electrodes will be placed above and under the eyes to record eye movements, as well as two electrodes on the chin to measure muscle tone. An electrocardiogram will also be recorded by placing two electrodes under each clavicle. In order to detect potential sleep apneas or hypopneas, thoracic and abdominal belts will be placed to record respiratory movements, nasal and oral thermistors to measure respiratory airflow and a finger pulse oximeter to measure oxygen saturation. All these sensors will be connected to a Siesta sleep system (Compumedics). Electrodes will be placed by an EEG technician.

### Statistical analysis

This study was designed to control an error risk α of 0.05 and a power of 80%. Assuming cognitive decline for 30% of BC women under HT versus 10% among healthy controls [50], the required sample size is 24 patients per group. We thus planned to enrol a total of 75 participants (25 BC patients receiving HT, 25 BC patients without indication to HT and 25 healthy women).

Descriptive statistics (relative frequency, mean value and SD) will be estimated for the socio-demographic and clinical variables from the total population included in the study. Comparison of means for sub-groups of participants will be realised by Student’s or Wilcoxon’s tests as appropriate. Comparisons of the proportions will be performed using both parametric and non-parametric tests. Link between PM and underlying cognitive functions, HT, and sleep quality, will be estimated thanks to univariate and multivariate logistic regression models, and results will be presented with odds ratios (OR) and their 95% confidence intervals (CI). All analyses will be conducted using R (version 3.4.1).

## Discussion

Numerous BC patients survivors report memory troubles. A recurring complaint is that they forget what they are supposed to do at the right moment, referring to PM. This PM is essential for self-sufficiency and medical adherence and thus the well-being of BC patients. Few studies have been published on PM functioning in BC patients, and PM process impairment needs to be more precisely evaluated. Coupled with PM impairment, several BC patients report sleep disturbances. Few studies have used PSG to assess sleep in BC, but results have revealed a decrease of sleep quality in BC patients. Knowing the involvement of sleep in memory consolidation, these results support the necessity to investigate the link between memory impairment and sleep disturbances in BC. Furthermore, HT seems to have a negative impact on memory and sleep, but there is still a lack of research in this area.

The current study will assess PM using an innovating and ecological virtual reality based task. The different types of components and intentions will be distinguished and correlated with performances in various neuropsychological tests. These tests will assess working memory, episodic memory, and executive functions known to be involved in PM functioning. The findings are expected to improve understanding of PM impairment and underlying mechanism in BC patients. Sleep will be assessed by PSG in order to obtain additional information related to sleep architecture. Sleep architecture parameters will also be correlated to PM scores and performances at night-time and day-time sessions will be compared in order to put forward the consolidation of prospective memories during sleep. These various assessments will be performed among BC patients treated or not with HT and healthy women, so that it will be possible to bring to light the possible specific impact of either BC and/or HT on PM functioning and sleep quality.

Even though the current study aims at assessing PM using an ecological task in a virtual environment, it remains a limitation. Virtual reality may provoke uncomfortable sensations like nausea and dizziness. Session of familiarisation will allow participants to become accustomed with virtual reality. If at the end of the session 1, participants are not used to virtual reality and feel sick, the task will be stopped and they will be withdraw from the study.

## Conclusion

The PROSOM-K trial is expected to bring multifaceted information on PM difficulties among BC patients. Indeed, the assessment of the relationship between components and intentions of PM using virtual reality, and neuropsychological tests, should lead to advanced knowledge about the PM processes impaired in BC patients. PSG data will be an opportunity to better explore the architectural sleep disturbances in BC. Thanks to the three groups of participants and the three sessions, some information should be given on the influence of sleep and HT on PM functioning. In a longer term, we expect our findings will be useful for patients and clinicians to understand and take into account their complaints, and thereby improve their take care. We also expect our results will further help to develop applications to improve their daily life and compensate for their PM impairment, as well as to improve cognitive therapy related to sleep and cognitive disorders.

## Additional files

Additional file 1: **Figure S1.** Subject during the PM task, in the immersive room (CIREVE, Caen). (JPG 1015 kb)

Additional file 2: **Figure S2.** PROSOM-K procedure. (PNG 503 kb)

## Abbreviations

BC: Breast Cancer
EB: Event-Based
HT: Hormonal Therapy
PM: Prospective Memory
PSG: Polysomnography
TB: Time-Based
