# Supplementary figures and images for: Impact of breast cancer on prospective memory functioning assessed by virtual reality and influence of sleep quality and hormonal therapy: PROSOM-K study
Source: BMC Cancer. 2018 Sep 3;18:866. doi: 10.1186/s12885-018-4762-2 (PMC6122719; doi:10.1186/s12885-018-4762-2)

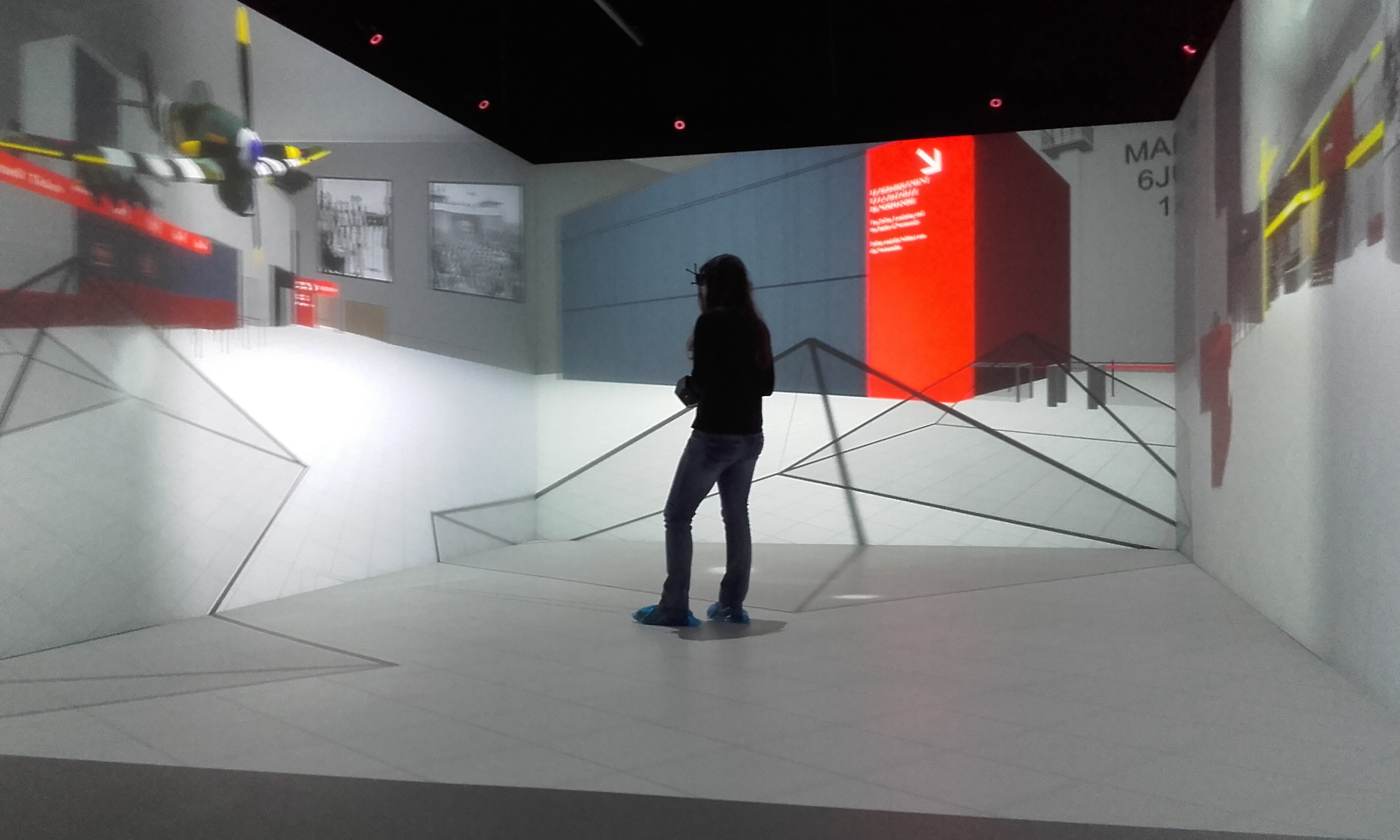

Supplement: Supplementary file 1 — Figure S1. Subject during the PM task, in the immersive room (CIREVE, Caen). (JPG 1015 kb) [file 12885_2018_4762_MOESM1_ESM.jpg]

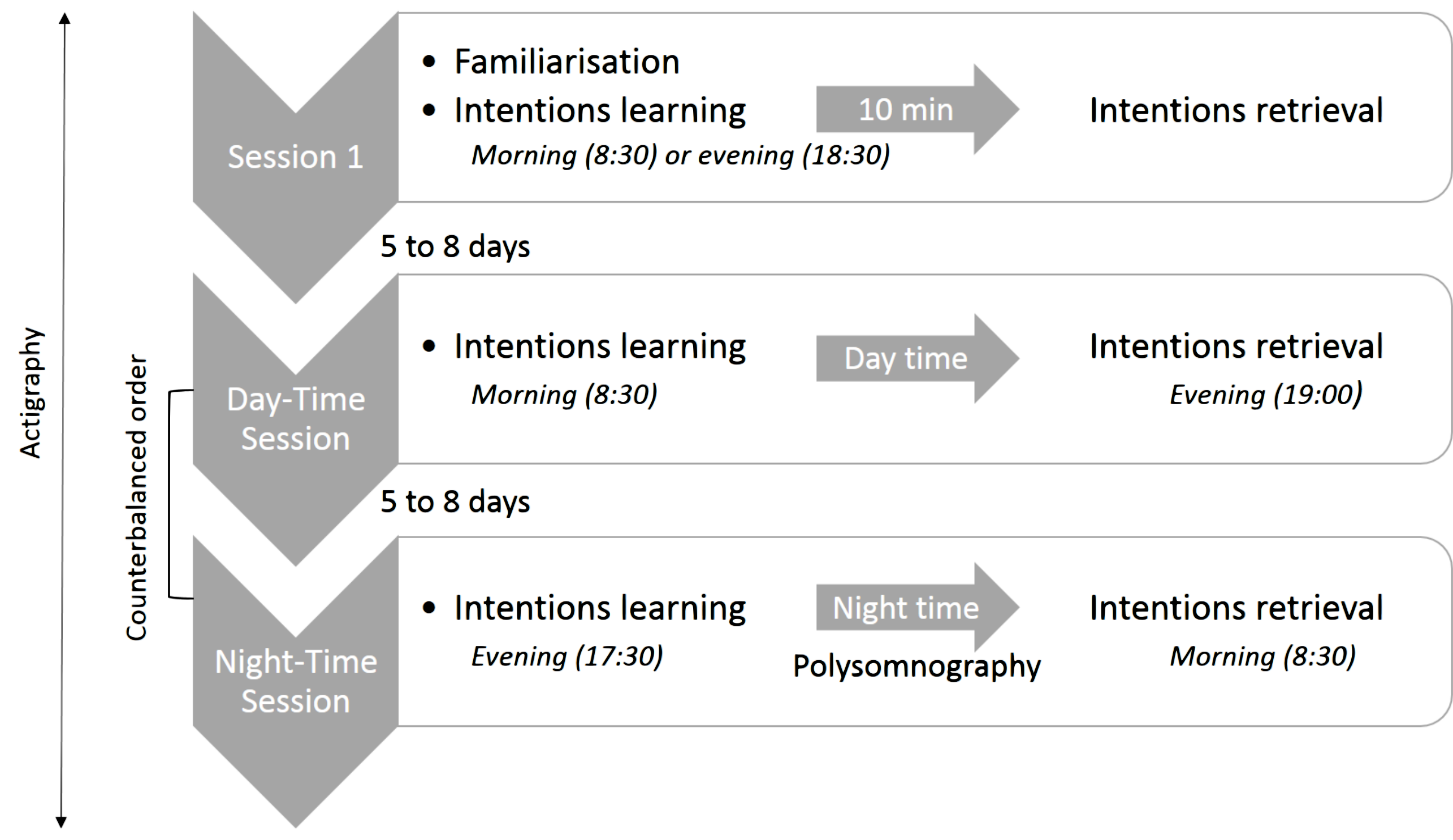

Supplement: Supplementary file 2 — Figure S2. PROSOM-K procedure. (PNG 503 kb) [file 12885_2018_4762_MOESM2_ESM.png]
